# Chronic pain among older adults and its impact on satisfaction with social participation: development and validation of the “Instrument to Assess Older Adults’ Social Participation”. A descriptive quantitative study

**DOI:** 10.1590/1516-3180.2022.0217.R1.310523

**Published:** 2023-07-31

**Authors:** Gabriela Costa Mastela, Júlia de Carvalho Galiano, Ligia Cangussu Tomaz Garcia, Maria Carolyna Fonseca Batista Arbex, Naira de Fatima Dutra Lemos, Fania Cristina Santos

**Affiliations:** I MD. Doctor and Volunteer Preceptor, Discipline of Geriatrics and Gerontology, Pain and Osteoarticular Diseases Service, Universidade Federal de São Paulo (UNIFESP), São Paulo (SP), Brazil.; II MD. Doctor and Volunteer Preceptor, Discipline of Geriatrics and Gerontology, Pain and Osteoarticular Diseases Service, Universidade Federal de São Paulo (UNIFESP), São Paulo (SP), Brazil.; III MD. Doctor and Volunteer preceptor, Discipline of Geriatrics and Gerontology, Universidade Federal de São Paulo (UNIFESP), São Paulo (SP), Brazil.; IV MSc. Doctor, Medical Coordinator, General Geriatrics and Memory Outpatient Clinic, Universidade de Araraquara (UNIARA), Araraquara (SP), Brazil; and Palliative Care Specialist, Universidad del Salvador, Pallium, Buenos Aires.; Universidad del Salvador, Buenos Aires, Argentina; V PhD. Social Worker, Affiliate Professor, Discipline of Geriatrics and Gerontology, Universidade Federal de São Paulo (UNIFESP), São Paulo (SP), Brazil.; VI PhD. Doctor and Affiliate Professor, Discipline of Geriatrics and Gerontology, Pain and Osteoarticular Diseases Service, Universidade Federal de São Paulo (UNIFESP), São Paulo (SP), Brazil.

**Keywords:** Aged, Social participation, Chronic pain, Quality of life, Older adults, Pain impacts, Social aspects

## Abstract

**BACKGROUND:**

We aimed to develop and validate a practical instrument to assess older adults’ satisfaction with their social participation (SP).

**DESIGN AND SETTING:**

This methodological validation study was conducted at a public higher education institution.

**METHODS:**

A two-phase study was designed, developed, and validated to assess older adults’ satisfaction with their SP. In the first phase, we conceptualized SP and developed an “instrument to assess older adults’ satisfaction with their SP (IAPSI),” as approved by a committee of specialists, pre-tested, and partially validated. Second, we determined the IAPSI’s reproducibility using Cronbach’s alpha to measure internal consistency, Pearson’s and Spearman’s coefficients to measure correlations, the Bland-Altman plot and intraclass correlation coefficient (ICC) to measure reproducibility. We also generated a receiver operating characteristic (ROC) curve.

**RESULTS:**

102 older adults (mean age, 87.29) participated in the first phase. Moderate internal consistency (Cronbach’s alpha 0.7) and significant moderate correlations with quality of life by World Health Organization Quality of Life (WHOQOL)-bref and by WHOQOL-old social domains (Pearson’s coefficients 0.54 and 0.64, respectively; P < 0.001) were found. The ROC curve indicated an IAPSI score of 17 as the threshold for the impact of pain on satisfaction with SP (83.3% sensitivity and 88.9% specificity, P < 0.001). In the second phase, 56 older adults (between 81 and 90 years old) participated. We found adequate intra- and inter-observer reproducibility for the IAPSI (ICC 0.96 and 0.78, respectively).

**CONCLUSION:**

We have developed a practical instrument with appropriate psychometric properties to assess older adults’ satisfaction with their SP.

## INTRODUCTION

Population aging is a worldwide reality, with several factors able to prevent this process from unfolding in an active and healthy way. The presence of pain can affect the physical, psychological, and social functions of older adults as well as their quality of life and must be recognized as a relevant problem for these individuals.^1^

Chronic pain is highly prevalent among older adults, affecting nearly 50% of those who live in the community and 80% of those who live in long-stay institutions.^2^ Because this prevalence is so high, numerous severe and potentially debilitating consequences develop in the aging process in addition to greater health care expenses.^3^

Therefore, it is necessary to acquire knowledge about the impact of chronic pain among older adults. Consequently, it is important to consider the social aspects of these effects. A bidirectional relationship between chronic pain and social participation has been reported. The presence of chronic pain was found to have a negative impact on various social aspects, with these aspects also having a negative impact on pain, both resulting in unfavorable health consequences.^4^

Social participation during the aging process is a crucial topic and should be highly encouraged given that the current concept of health goes beyond questions regarding “diseases”.^5^

The scientific literature has not yet provided a well-defined consensus on older adults’ social participation, which is a complex multidimensional process. Some authors argue that, from a social psychology perspective, individuals’ social participation should conceptually include the time dedicated to social experiences and the time spent in the presence of others.^6^

If we consider the impact of chronic diseases and their treatments on quality of life, measuring older adults’ degree of satisfaction with their social participation can provide relevant information.^7^ Some instruments have already been proposed for such measures, but they derive mainly from a health perspective and address domains of self-care and mobility.^6^ The Patient-Reported Outcomes Measurement Information System is one example, and there is already a Brazilian version with sets of items related to “Satisfaction with Participation in Social Roles” (14-items) and “Satisfaction with Participation in Discretionary Social Activities” (12-items). Although comprehensive, these methods do not offer clinical practicality.^8^

Thus far, we have not found an available measuring instrument throughout the extant body of literature that assesses social participation exclusively among the older adult population or any tools that assess these individuals’ satisfaction with their social participation. We considered the possibility of assessing older adults’ satisfaction with their social participation and applied this assessment to older adults with chronic pain.

## OBJECTIVE

We aimed to develop and validate a construct for this purpose by presenting a measuring instrument to assesses older adults’ satisfaction with social participation. This will allow us to understand the potential impact of chronic pain on their (that of older individuals) satisfaction with social participation.

## METHODS

**Figure 1:**
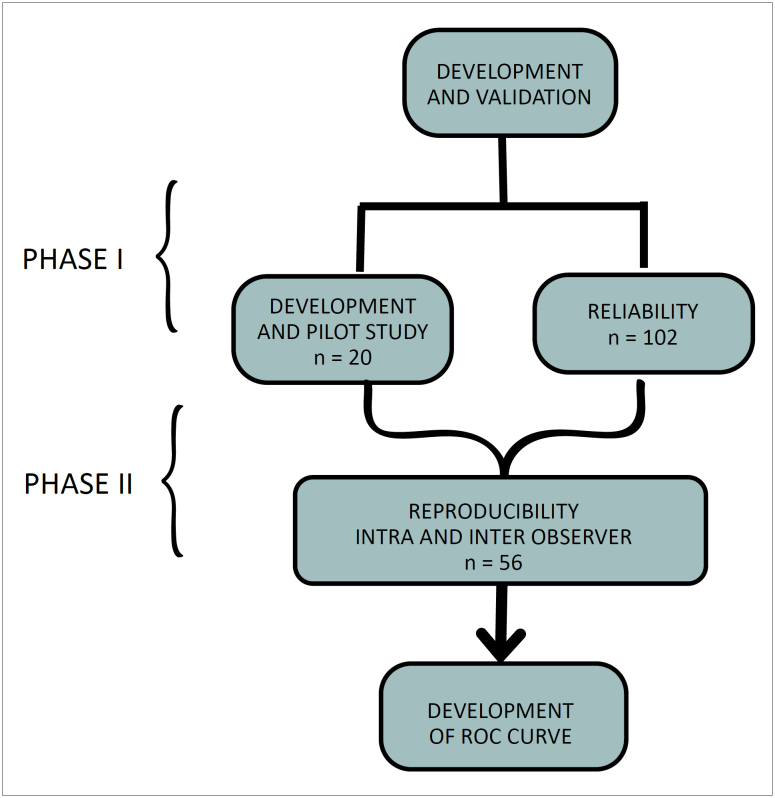
Diagram of the manufacturing process of development and validation.

This study has been methodologically validated. The methodology involved validating a prepared instrument and was conducted in two stages with approval from a Research Ethics Committee (Figure 1): **stage one** – the development and assessment of the reliability of a measuring instrument to assess older adults’ satisfaction with their social participation (Certificate of Presentation of Ethical Appreciation:05444918.0.0000.5505, approval date December 4, 2021); **stage two** – an assessment of the reproducibility of the instrument to assess older adults’ satisfaction with their social participation (IAPSI) among older adults with chronic pain (Certificate of Presentation of Ethical Appreciation:26467219.7.0000.5505, approval date January 16, 2020).

### Developing the instrument

We developed a construct based on the handbook of procedures for “developing measuring instruments,” suggested by Kline.^9^ This handbook refers to three phases:1) Theoretical foundation – this instrument was based on a narrative bibliographic review of the topic “older adults’ satisfaction with their social participation”; 2) Formulation of items for a simple construct – a process that involved preparing questions related to satisfaction with social participation in the aging process, which should include terms understood by older adults; 3) Preliminary analysis of the difficulties in understanding the formulated questions – a phase that required the participation of invited judges, i.e., a consensual judgment by a committee of experts on the subject matter was necessary, which was formed by six experienced specialists in different areas of health, namely social work, nursing, physical therapy, psychology, geriatrics, and algology.

For the theoretical foundation phase, we searched the LILACS and MEDLINE databases for publications in Portuguese and English using the index terms “social participation” and “older adult” over the preceding ten years. We also searched for references to chronic pain and its social aspects in older adults.

To formulate the items for the intended construct, we selected topics that had the best potential to translate “satisfaction with social participation among older adults” that could also have the potential to interact with the presence of pain. Thus, we developed a comprehensive questionnaire with short answers based on a 5-point Likert-type scale (“very dissatisfied”, “dissatisfied”, “neither satisfied nor dissatisfied”, “satisfied”, and “very satisfied”).

The committee of invited specialists judged several aspects of the construct under development, covering aspects of: clarity, representativeness, and comprehensiveness of the questions; formatting of the established items; instructions regarding possible answers; and selection of the construct’s main domains. After the judging committee pre-approved this construct, a pretest was conducted.

For the pre-test phase, we randomly selected 20 participants of both sexes who were 60 years or older and who were receiving care at a geriatrics and gerontology outpatient unit that provides public services in the city of São Paulo. All the participants provided written informed consent.

We assessed participants’ potential difficulties in understanding the questions and their answers. After the pre-test phase, the judging committee issued another opinion for the final approval of the “Instrument to Assess Older Adults’ Social Participation” (IAPSI) (**Appendix 1**).

We established that the items for the IAPSI should be arranged in four major domains of older adults’ social participation: domestic life (household chores), community life (community events and means of transportation), interpersonal relationships (friends, family), and free time (spare time after satisfying all needs, leisure activities, and hobbies). We also determined that the construct would have only five items (one for domestic life, two for community life, one for interpersonal relationships, and one for free time) and that each would have five response options (a total of 25 responses).

With the judging committee’s final approval, we have already obtained an important type of validity for a measuring instrument, “content validity,” which is related to evaluating a measuring tool’s representativeness with respect to the universe of content.^10^

### Reliability of the Measuring Instrument

The psychometric property of reliability refers to the consistency of a construct, which can be of three types: internal consistency (correlation between items), reproduction with a test-retest by the same observer (intraobserver reproducibility), and reproduction involving different observers (interobserver reproducibility).^11^

### Internal consistency assessment and validation

For these assessments, we randomly recruited adults of both sexes who were 60 years old or older and who were receiving care at a geriatrics and gerontology outpatient unit that provides public services in São Paulo. We adopted non-probability, casuistic, convenience sampling, involving individuals who wanted to participate in the study.

Those selected met the established criteria, and all signed an Informed Consent Form. The inclusion criterion required that participants receive regular follow-up care at the aforementioned outpatient unit. The exclusion criteria included older adults who presented with cognitive decline, as defined by a score in the Mini-Mental State Examination that is below the expected score for the individual’s level of education, or with neoplasm-related pain, or who had been hospitalized in the last three months.

A semi-structured questionnaire with sociodemographic (age, sex, race/ethnicity, and marital status) and clinical data was administered individually. The latter data referred to older adults’ personal perception of their health (“excellent”, “good”, “regular”, and “bad”), presence of chronic pain (duration of six months or more), and pain intensity according to the verbal numerical rating scale (vNRS) (classification:1–3 for mild pain; 4–6 for moderate pain; and 7 or more for severe pain).

At the same time, we applied two instruments to assess older adults’ functionality in daily living, the Katz and Lawton scales, which relate to capabilities in basic and instrumental activities of daily living, respectively, and two instruments that are widely used to assess quality of life, the World Health Organization Quality of Life (WHOQOL)-bref and WHOQOL-old (only the social participation domain of the latter).^12,13^ Finally, we applied the IAPSI and recorded its application time.

This process allowed us to evaluate the internal consistency of IAPSI and to obtain its “criterion validity,” an “operationally defined” property, one of the most crucial steps in the validation of measuring instruments. This refers to the degree in which an instrument’s operationalization is similar to others, stipulating that they should be similar.^11^ This type of validity involves comparisons between the measuring instrument and a “gold standard” assessment. However, when such comparisons cannot be made, sometimes as a result of the absence of a gold standard, routine clinical parameters are used. Here, we obtained convergent criterion validity based on the correlations between the IAPSI and quality of life according to the WHOQOL-bref and the social domain of the WHOQOL-old.

### Reproducibility assessment

This assessment was performed in the second stage of the study, and the sample size was calculated by considering a maximum sampling error of 10% (ideally, it would be less than 5%; however, we considered the difficulties in data collection and the application of the instruments, especially the application of the IAPSI, which should be applied on two different days). With a 95% confidence level, we considered two aspects: the estimated number of older adults who regularly received care at the geriatrics and gerontology outpatient clinic at the Universidade Federal de São Paulo (approximately 1,600 patients) and the prevalence of chronic pain among these individuals, which would be approximately 20% according to the international literature and an observational study conducted in the aforementioned outpatient unit,^14,15^ and determined a sample size of 55 participants.

In this phase, we initiated a new random recruitment of older adults of both sexes who were 60 years or older and who had received care at the same outpatient unit in the first phase of the study. The inclusion criteria were participants who experienced chronic pain (duration of six months or more) of different etiologies with a minimum intensity of three, according to the vNRS, and were motivated to participate in this stage, which required their involvement in assessments on two different days. All the participants signed an Informed Consent Form. We excluded those who presented with cognitive decline, as defined by a score on the Mini-Mental State Examination that is below the expected score for the individual’s level of education, or with neoplasm-related pain or those who had been hospitalized in the last three months.

We gathered demographic (age, sex, and race/ethnicity) and clinical data and referred to pain based on its intensity using the vNRS and its multidimensionality using the “Geriatric Pain Measure” (GPM). The latter instrument exclusively considers sensory-discriminative, affective-motivational, and cognitive-evaluative aspects of pain in older adults (classification: mild pain – 1 29; moderate – 30 69; severe – 70 100).^16^

To determine IAPSI’s reproducibility of the IAPSI, we applied it three different times: on two different occasions in the initial assessment by two trained interviewers who made separate assessments (inter-observer reproducibility) and after 15 days, when the participants returned for another application of the IAPSI by only one of the interviewers involved (intra-observer reproducibility).

### Statistical analysis

IBM SPSS Statistics version 17 (Chicago, United States) and Microsoft Excel 2010 (Washington, United States) were used for the data analysis. Quantitative (mean and standard deviation) and qualitative variables were examined according to the Equality of Two Proportions tests. We determined IAPSI’s internal consistency via Cronbach’s alpha, and its associations with pain (vNRS and GPM), quality of life (WHOQOL-bref), and the social domain (WHOQOL-old) using Pearson’s coefficient. We used Spearman’s correlation for the correlations between the IAPSI and pain (ENV), WHOQOL-bref, and the social domain of the WHOQOL-old. We created a receiver operating characteristic (ROC) curve with the data from stage one of the study based on pain (intensity) and the cut-off point for overall quality of life of less than 60 by the WHOQOL-BREF, which demonstrated excellent sensitivity and a negative predictive value for the screening of older adults who probably had a worse quality of life.^17^ We used the Bland-Altman plot and the intraclass correlation coefficient (ICC) to determine inter- and intra-observer agreement and established a 5% significance level.

## RESULTS

The development of the instrument culminated in a construct that was easily understood by older adults. Furthermore, the researchers found that the tool was easy and quick to apply. The average completion time was four minutes.

A total of 102 older adults participated in the first stage of the study, most of whom were female (74%), white (60%), and widowed (63%). Moreover, the vast majority of participants were functionally independent for basic (98%) and instrumental (56%) activities of daily living (Table 1).

**Table 1:** Characteristics of the participants in stage one of the study

Characteristics		n	%	Mean	SD	Interval
**Age**		102		87.29	4.37	80–101
**Sex**						
	Female	75	74			
	Male	27	26			
**Race/Ethnicity**						
	Black	1	1			
	Asian	19	19			
	White	61	60			
	Other	21	20			
**Marital status**						
	Married	29	28			
	Divorced	5	5			
	Single	4	4			
	Widowed	64	63			
**BADL**						
	Independent	100	98			
	Partial dependence	1	1			
	Total dependence	1	1			
**IADL**						
	Independent	57	56			
	Mild dependence	38	37			
	Moderate dependence	6	6			
	Severe dependence	1	1			
**Personal perception of health**						
	Bad	2	2			
	Regular	38	37			
	Good	44	43			
	Excellent	18	18			
**IAPSI**				18.59	2.69	12–25
**WHOQOL-bref**						
	Physical domain			66.53	18.39	17.9–100
	Psychological domain			73.41	14.21	29.2–100
	Social domain			69.61	12.33	25–100
	Environmental domain			65.95	12.47	18.8–96.9
	Overall			68.89	10.74	33.4–91.4
**WHOQOL-old/Social**				15.04	2.69	8–24
**vNRS**				6.1	2.39	2–10

BADL = Basic activities of daily living; IADL = Instrumental activities of daily living; IAPSI = Instrument to Assess Older Adults’ Social Participation; WHOQOL-bref/old = World Health Organization Quality of Life-brief/old; vNRS = Verbal Numerical Rating Scale; SD = standard deviation.

Chronic pain affected approximately 60% of the participants and its intensity was mostly moderate (mean vNRS, 6.1) (Table 1).

Regarding quality of life, according to the WHOQOL-bref, we found a higher mean in the psychological domain than in the other domains (score = 73.41), but the difference was not statistically significant. For the social domain of the WHOQOL-old, we observed a mean of 15.04 (Table 1).

Based on Cronbach’s alpha, the IAPSI’s internal consistency was moderate (approximately 0.7) (Table 2).

**Table 2:** IAPSI’s internal consistency based on Cronbach’s alpha

IAPSI	Correlation between items
Item 1	0.452
Item 2	0.552
Item 3	0.688
Item 4	0.629
Item 5	0.494
**Total Cronbach’s alpha**	0.689

IAPSI = Instrument to Assess Older Adults’ Social Participation.

Overall, there was a significant correlation between IAPSI and quality of life by WHOQOL-bref and each of its domains, according to Pearson’s coefficient (environment 50%, social 45%, psychological 40%, physical 31%, overall 54%; P < 0.001). We also found a significant correlation between the IAPSI and the social domain of the WHOQOL-old (64%; P < 0.001). We found adequate convergent criterion validity for the IAPSI.

An ROC curve was used to determine the cut-off score for the IAPSI, indicating the impact of chronic pain on older adults’ satisfaction with their social participation. Scores less than or equal to 17.5, with 83.3% sensitivity and 88.9% specificity, indicated impact of chronic pain on older adults’ satisfaction with their social participation (P < 0.001).

In the second stage of the study, we obtained a sample of 56 older adults who were mostly female (89.3%) and white (62.5%). Additionally, most patients presented with moderate chronic pain according to the vNRS (mean intensity: 6) and GPM (mean score: 66.1). For the IAPSI, we observed a mean total score of 17.37 (ranging from 10 to 24) (Table 3).

**Table 3:** Characteristics of the participants in stage two of the study

Characteristics		n	%	Mean	SD	Interval
**Age (years)**						
	60–70	13	23.2			
	71–80	14	25.0			
	81–90	26	46.4			
	91–100	2	3.6			
	> 100	1	1.8			
**Sex**						
	Female	50	74			
	Male	6	26			
**Race/Ethnicity**						
	Black	5	1			
	Asian	1	19			
	White	35	60			
	Other	15	20			
**GPM**				66.10	20.24	8–99
**vNRS**				6	2	1–9
**IAPSI**				17.37	3.54	10–24

GPM = Geriatric Pain Measure; vNRS = verbal numerical rating scale; IAPSI = instrument to assess older adults’ social participation; SD = standard deviation.

There was an inverse correlation between IAPSI and pain, with greater pain corresponding to lower IAPSI scores. Thus, as pain worsens, older adults’ satisfaction with their social participation lowers (Spearman’s coefficient, -0.282; P < 0.004).

**Figure 2:**
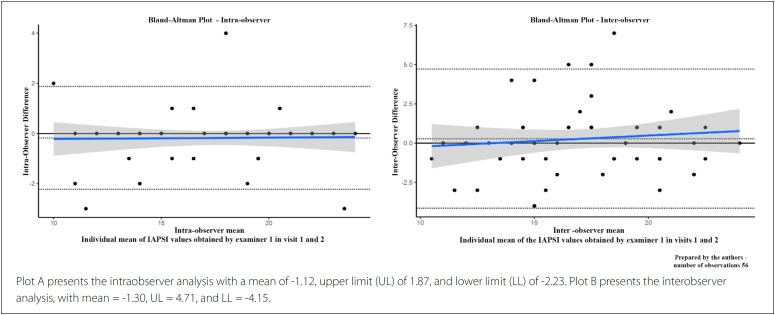
Bland-Altman plots.

A comparison of the total scores between the application and reapplication of IAPSI by the same observer did not result in any significant difference (17.38 ± 3.54 versus 17.55 ±3.53; P = 0.79), and the intra-observer ICC was 0.95. A comparison of the IAPSI scores by the two examiners of the study did not result in a significant difference either (17.38 ±3.54 versus 17.09 ± 3.33; P = 0.66), and the inter-observer ICC was 0.78. The Bland-Altman plot indicated good agreement between the IAPSI scores obtained by the two examiners. The results were the same when comparing scores obtained by the same observer (Figure 2).

## DISCUSSION

Currently, there are no practical, standardized measuring instruments to clinically assess social participation in the aging process^6^ or instruments for measuring older adults’ satisfaction with their social participation, especially those with chronic pain.

The construct presented herein is the first to be developed for the purpose of approaching older adults’ satisfaction with their social participation. The IAPSI is simple and quick to apply and older adults understand it well. It considers older adults’ satisfaction with their social participation, and “social participation” has been considered one of the pillars of healthy aging.^18^

In preparing the intended construct, we tried to include important aspects of social participation by faithfully following the guide provided by Kline^9^ for developing instruments. We also attempted to obtain adequate validation, which is important for measuring instruments.

Regarding the internal consistency of the IAPSI, a property related to the reliability of the measuring instruments, we found that it was moderate according to Cronbach’s alpha (coefficient 0.7).

In the first stage of the study, there was a significant correlation between the IAPSI and the presence and intensity of chronic pain. We observed the same occurrence in the second stage of the study, when we found a correlation between the IAPSI and chronic pain based on its multidimensionality using the GPM. Decades since Tollison^19^ described the complex phenomenon of pain and emphasized an adequate assessment of its various dimensions in approaches to patients’ pain conditions: physiological (semiological characteristics, among others), sensory (intensity, quality), affective (anxiety, depression), cognitive (meaning of pain, adaptive resources), behavioral (pain behavior, medication acceptance), psychosocial (interpersonal interaction, social and family life, interrelation with home/work, leisure), and sociocultural (ethnocultural, environmental factors). Pain was assessed in this manner. The social aspects of individuals with pain have long been considered important but have rarely been addressed.

There was a significant correlation between the IAPSI and quality of life, according to the WHOQOL-bref (all domains), and between the IAPSI and the social domain of the WHOQOL-old (all correlations; P < 0.001). Recently, Ferreti et al.^20^ noted that quality of life by WHOQOL-old changed in accordance with the presence or absence of pain, and that the social participation domain was one of the most affected in this evaluation. Celich and Galon (2009)^21^ also observed that chronic pain among older adults was a limiting factor in their daily activities (going to church, dancing, and participating in community activities), restricting their social life and resulting in a negative perception of their quality of life. Therefore, approaches to aging are essential.

Regarding IAPSI reproducibility (inter- and intra-observer), we found strong inter- and intra-observer agreements according to the Bland-Altman plots (0.78 and 0.96 for inter- and intra-observer, respectively). Thus, an additional valid psychometric property was observed for the IAPSI.

Moreover, we evaluated the IAPSI’s performance according to sensitivity and specificity indicators and constructed an ROC curve to determine the best cutoff point to assess the satisfaction of older adults with chronic pain with their social participation. With 83.3% sensitivity and 88.9% specificity, scores lower than or equal to 17.5 indicated the impact of chronic pain on older adults’ satisfaction with their social participation (P < 0.001).

This study presented limitations, such as the IAPSI’s moderate internal consistency, which might even suggest that it contains items that can be excluded. However, the items evaluated were essential for social participation and were thus maintained in the construct. This weakness may be acceptable, as the instrument aims to measure different characteristics of social participation. Another limitation is that we assessed IAPSI in a population of considerably older and more functionally independent adults, which is interesting in a way, as it allowed for an early assessment of impacts that may impair functional capacity in the aging process.

Due to the importance and practicality of the IAPSI in assessing the impact of pain on older adults’ satisfaction with their social participation, we suggest including this instrument in clinical protocols and research on approaches to pain during the aging process.

## CONCLUSION

In conclusion, the IAPSI is a proposed instrument for assessing older adults’ satisfaction with their SP, especially for those with chronic pain. This construct was very simple and quick to apply, and demonstrated satisfactory measurement properties, such as internal consistency, reproducibility, content, and criterion validity.

## Appendix

**Appendix 1:** Instrument to assess older adults’ social participation

Instrument to Assess Older Adults’ Social Participation - IAPSI						
Please answer the following questions in reference to the last two weeks. If you are not sure about which answer to give, please choose the alternative that seems most appropriate to you.						
**Domestic Life**	1 – How satisfied are you with your domestic activities?	Very dissatisfied	Dissatisfied	Neither satisfied nor dissatisfied	Satisfied	Very satisfied
		1	2	3	4	5
**Community Life**	2– How satisfied are you with your participation in community events (mass, worship, fairs…)?	Very dissatisfied	Dissatisfied	Neither satisfied nor dissatisfied	Satisfied	Very satisfied
		1	2	3	4	5
	3 – How satisfied are you with your means of transportation?	Very dissatisfied	Dissatisfied	Neither satisfied nor dissatisfied	Satisfied	Very satisfied
		1	2	3	4	5
**Interpersonal Relations**	4 – How satisfied are you with your personal relationships (friends, family, acquaintances…)?	Very dissatisfied	Dissatisfied	Neither satisfied nor dissatisfied	Satisfied	Very satisfied
		1	2	3	4	5
**Free Time**	5– How satisfied are you with the use of your free time (leisure, hobbies…)?	Very dissatisfied	Dissatisfied	Neither satisfied nor dissatisfied	Satisfied	Very satisfied
		1	2	3	4	5

**Domestic life: (1) ________**

**Community life: (2+3) _______**

**Interpersonal relations: (4) _______**

**Free time: (5) ______**

**Total score: ______ (sum of the points for each item based on the answers given)**

**Note:** A higher score corresponds to a higher degree of satisfaction with social participation among older adults. Suggested cut-off score £ 7 for very dissatisfied.

## References

[1] AGS Panel on Persistent Pain in Older Persons (2002). The management of persistent pain in older
persons. J Am Geriatr Soc.

[2] Almeida CBL, Félix RH, Cendoroglo MS, Santos FC (2017). Pain-induced depression in the elderly: Validation of
psychometric properties of the Brazilian version of the “Geriatric Emotional
Assessment of Pain” – GEAP-b. Rev Assoc Med Bras (1992).

[3] American Geriatrics Society Panel on Pharmacological Management of
Persistent Pain in Older Persons (2009). Pharmacological management of persistent pain in older
persons. J Am Geriatr Soc.

[4] Dueñas M, Ojeda B, Salazar A, Mico JA, Failde I (2016). A review of chronic pain impact on patients, their social
environment and the health care system. J Pain Res.

[5] Rebellato C (2016). Preditores da Participação Social de idosos independentes cadastrados em
Estratégias de Saúde da Família do município de Araras/SP [thesis].

[6] Gorjão S (2011). Envelhecimento Ativo: O papel da participação social Construção e
Validação de um Instrumento [dissertation].

[7] Silva e Costa ZMS, Pinto RMC, Mendonça TMDS, Silva CHMD (2020). Validação brasileira dos bancos de itens Distúrbio do Sono e
Distúrbio da Vigília do Patient-Reported Outcomes Measurement Information
System (PROMIS) [Brazilian validation of the item banks on Sleep Disturbance
and Wake Disturbance in the Patient-Reported Outcomes Measurement
Information System (PROMIS)]. Cad Saude Publica.

[8] Silva MCL (2019). Validação e calibração da versão brasileira do domínio satisfação com a
participação social do Patient-Reported Outcomes Measurement Information
System - PROMIS® - (versão 1.0) [thesis].

[9] Kline P (1995). The handbook of psychological testing.

[10] Maack DJ, Buchanan E, Young J (2015). Development and psychometric investigation of an inventory to
assess fight, flight and freeze tendencies: the fight, the flight, freeze
questionnaire. Cogn Behav Ther.

[11] Ferraz AS (2016). Psicometria. Aval Psicol.

[12] (1998). The World Health Organization Quality of Life assessment
(WHOQOL): development and general psychometric properties. Soc Sci Med.

[13] Power M, Quinn K, Schmidt S, WHOQOL-OLD Group (2005). Development of the WHOQOL-old module. Qual Life Res.

[14] Thomas E, Peat G, Harris L, Wilkie R, Croft PR (2004). The prevalence of pain and pain interference in a general
population of older adults: cross-sectional findings from the North
Staffordshire Osteoarthritis Project (NorStOP). Pain.

[15] Almeida CBL, Félix RH, Cendoroglo MS, Santos FC (2017). Pain-induced depression in the elderly: Validation of
psychometric properties of the Brazilian version of the “Geriatric Emotional
Assessment of Pain” - GEAP-b. Rev Assoc Med Bras (1992).

[16] Silva PA, Soares SM, Santos JF, Silva LB (2014). Cut-off point for WHOQOL-bref as a measure of quality of life of
older adults. Rev Saude Publica.

[17] Rebellato C, Hayashi MCPI (2014). Participação social do idoso - estudo bibliométrico da produção
científica recente. RECIIS – Rev Eletron de Comun Inf Inov Saude.

[18] Tollison CD, Satterthwaite JR, Tollison JW (1994). Handbook of pain management.

[19] Ferretti F, Castanha A, Padoan E, Lutinski J, Silva M (2018). Quality of life in the elderly with and without chronic
pain. Braz J Pain.

[20] Celich KL, Galon C (2009). Dor crônica em idosos e sua influência nas atividades da vida
diária e convivência social. Rev Bras Geriatr Gerontol.

